# The top 100 most cited papers on endometrial carcinoma: A bibliometric analysis

**DOI:** 10.3389/fonc.2022.987980

**Published:** 2022-08-18

**Authors:** Peichen Xiao, Chenchen Yao, Guangxin Wang

**Affiliations:** ^1^ Department of gynecology, Jinan Central Hospital, Shandong University, Jinan, Shandong, China; ^2^ Shandong Innovation Center of Intelligent Diagnosis, Jinan Central Hospital, Shandong University, Jinan, Shandong, China

**Keywords:** endometrial carcinoma, bibliometric analysis, research hotspots, web of science core collection, VOSviewer

## Abstract

**Objective:**

This study aimed to analyze the top 100 most cited papers and research trends on endometrial carcinoma *via* bibliometric methods.

**Methods:**

On the 1^st^ of March 2022, the top 100 most cited papers regarding endometrial carcinoma published from 1971 to 2021 were identified through searching Web of Science Core Collection database and the following data: title, author, journal, publication year, country and institution were extracted. Microsoft Office Excel (2019) was used for descriptive statistical analysis. VOSviewer (1.6.18) was used to perform and visualize co-authorship analysis and co-occurrence analysis.

**Results:**

These 100 papers were cited a total of 45, 685 times, and the mean number of citations was 456.85 (range, 228 to 2487). Most papers were published between 1996 and 2000, and between 2006 and 2010. *The Lancet* published the largest number of papers (n=12), followed by *Gynecologic Oncology* (n=11). Most of the papers were from the United States (n=58), followed by Italy (n=8) and Netherlands (n=7). Duke University, Johns Hopkins University, University of California San Francisco and University of Southern California (all in United States) contributed the most papers (n=4, respectively). Nicoletta Colombo contributed the most papers (n=3) as the corresponding author. The co-occurrence keywords were classified into three clusters: cluster 1 (epidemiology study), cluster 2 (molecular biology study) and cluster 3 (clinical treatment study). Early research that was published prior to 2005 in this field was mainly focused on epidemiology and molecular biology; the mean publication year for keywords in cluster 3 was later than other clusters. The keywords “external-beam radiotherapy,” “uterine serous carcinoma,” and “intermediate-risk” showed relatively later mean publication year and lower mean frequency of occurrence.

**Conclusions:**

This study provides medical researchers with bibliometric information relating to endometrial carcinoma. Our results show that the United States is a clear leader in this field. The clinical treatment of endometrial carcinoma has received increasing levels of attention over recent years and is likely to remain a major area of research in the future. Meanwhile, it is recommended to pay attention to potential research hotspots, such as external-beam radiotherapy, uterine serous carcinoma and intermediate-risk.

## Introduction

Endometrial carcinoma, an epithelial tumor of the endometrium, is one of the three main malignant tumors of the female reproductive tract. The global incidence of endometrial carcinoma is increasing annual and the age of onset is becoming younger (1). According to global cancer statistics released in 2021, the number of new cases of endometrial carcinoma worldwide in 2020 was 420,000, ranking this disease sixth in terms of all female cancers (2). Thousands of papers on endometrial carcinoma have been published in the research areas of epidemiology, pathology, diagnosis, molecular biology and clinical treatment, making it challenging for researchers to identify the most influential papers, research hotspots and future directions in this field.

Bibliometric analysis is defined as a statistical evaluation of published scientific papers, books, or book chapters (3–6). The academic influence of a research article can be measured by the number of times it has been cited by other authors (7, 8). A detailed bibliometric analysis of the most cited papers would be very useful and facilitate our understanding of the future direction of development in this discipline (9). Bibliometric analysis is being used in multiple clinical specialties, including dermatology, cardiology and urology (10–12). In this study, we aimed to identify the top 100 most cited papers on endometrial carcinoma and analyze their bibliometric characteristics, thus identifying the research hotspots and future directions in this field.

## Methods

On the 1^st^ of March 2022, papers regarding endometrial carcinoma published from 1971 to 2021 were retrieved through search of the Web of Science Core Collection database. (https://www.webofscience.com/wos/woscc/basic-search). The literature retrieval strategy was as follows: (Title = endometrial neoplasm* OR endometrial carcinoma* OR endometrial cancer* OR endometrium carcinoma* OR endometrium cancer*). The time frame for publications was set from 1971 to 2021 as papers published prior to 1971 may have been outdated. The search was limited to articles or reviews and excluded other types of papers such as editorials, meeting abstracts, letters and news reports. We also excluded papers in which endometrial carcinoma was only listed in a group of diseases and papers that focused on other topics. We only included papers that had been written in English. Two investigators (PX and CY) independently screened the title, abstract and whole text of papers to identify the top 100 most cited papers relating directly to endometrial carcinoma. Next, the title, corresponding author, journal, citation count, publication year, impact factor (IF), country, institution and research type of the top 100 most cited papers were extracted. The country and institution affiliated with each of the included articles were recorded based on the corresponding author. Microsoft Office Excel (2019) was used for descriptive statistical analysis and IFs were defined according to the Journal Citation Report (2020).

VOSviewer (1.6.18) was used to perform and visualize co-authorship analysis and co-occurrence analysis of keywords (13). The visualization maps were generated by VOSviewer based on the scope of search terms described above. In the network visualization and overlay visualization maps, different nodes represented different terms such as authors and keywords, while the size of nodes represented the corresponding frequency of occurrence. The links between nodes represented co-occurrence relationships while the width of the links indicated the strength of correlation between the two nodes. In the density visualization maps, terms were distributed according to the mean frequency of appearance. Terms in red occurred with the highest frequency, followed by yellow, green, and cyan.

## Results

### Citation counts

Using our specific search terms, literature screening identified 14, 227 papers. The top 100 most cited papers are listed in **Supplementary Table S1**. When considering the top 100 most cited papers, we found that the number of citations ranged from 228 to 2487 with a total of 45, 685 citations. The mean number of citations was 456.85. Of the top 100 most cited papers, 24 papers were cited more than 500 times and 9 papers were cited more than 1000 times. The total number of citations for the top 10 most cited papers was 13, 222.

### Publication year and research focus

**Figure 1:**
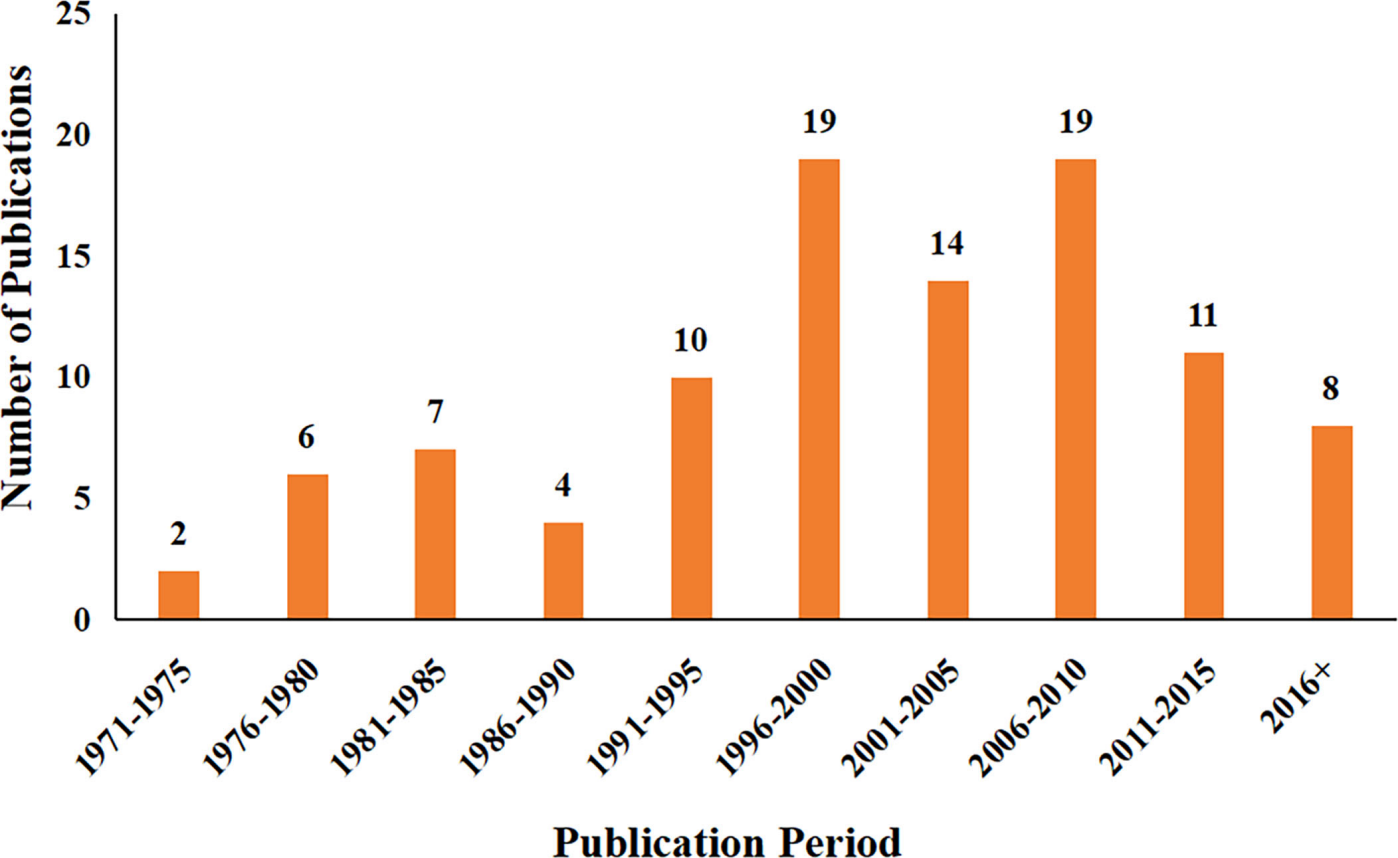
The number of papers published during each five-year interval between 1971 and 2021.

**Figure 2:**
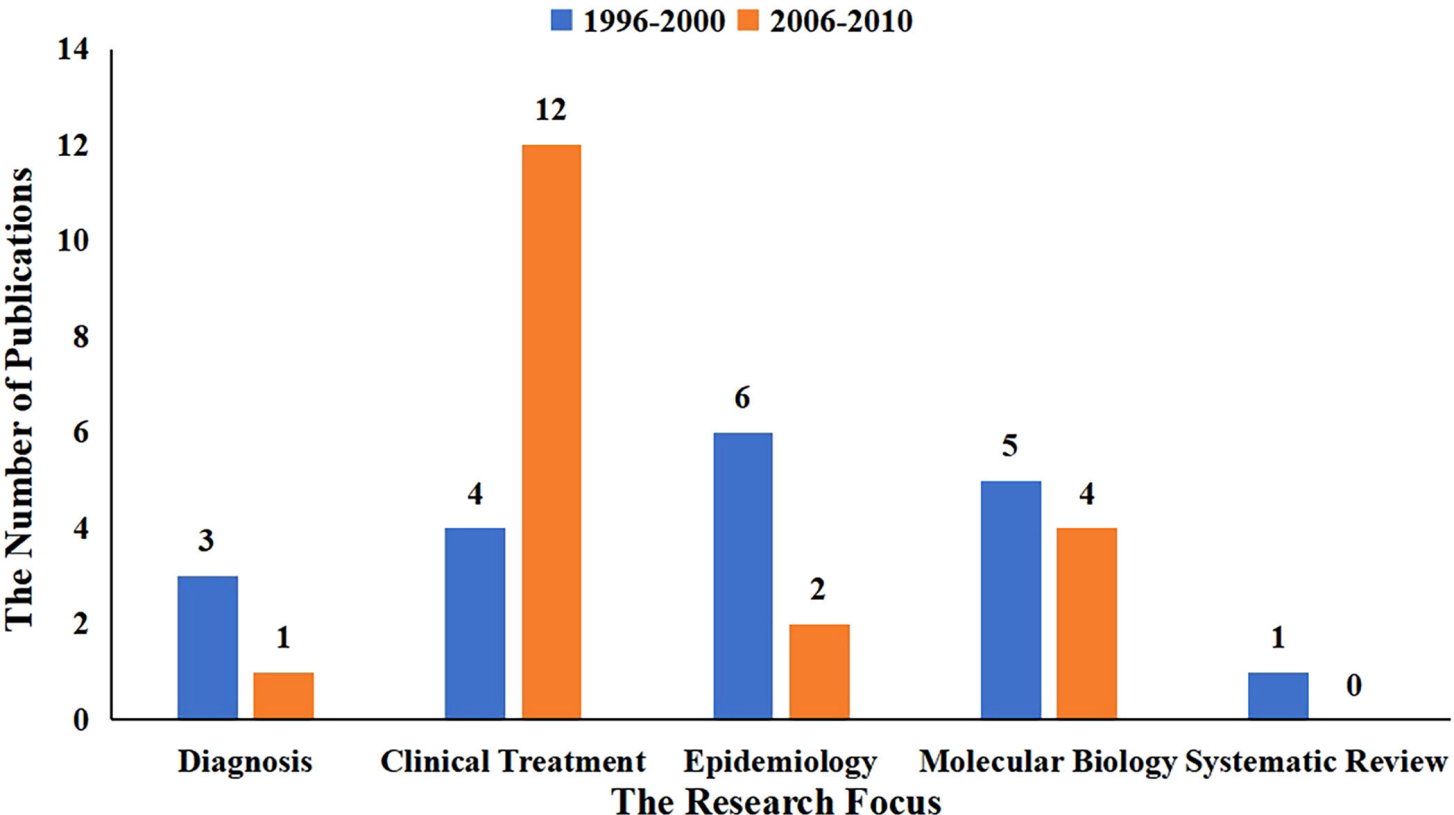
The research focus of papers published between 1996 and 2000 and between 2006 and 2010.

The year of publication for the top 100 most cited papers were divided into 10 five-year intervals (**Figure 1**). Most papers were published between 1996 and 2000, and between 2006 and 2010 (19 papers were published in both of the five-year intervals). In terms of research focus, these papers were divided into the following categories: diagnosis, clinical treatment, epidemiology, molecular biology and systematic reviews. **Figure 2** shows the research focus for papers published in the above two five-year intervals.

#### Research type

With regards to research type, the top 100 most cited papers were divided into five groups: (1) observational studies (OS) including epidemiology, case-control and cohort studies; (2) basic science studies (BS) including pathology, experimental and animal studies; (3) randomized controlled trials (RCTs); (4) clinical guidelines, and (5) review articles including meta-analysis and systematic reviews. The respective number of papers in each of these five groups were as follows: OS (n=34), BS (n=31), RCTs (n=14), review articles (n=18) and clinical guidelines (n=3).

#### Journals and impact factors

The top 100 most cited papers were published in 33 different journals. We ranked the journals with more than one paper and their impact factors (2020/last five years) in descending order by the number of papers published (**Table 1**). *The Lancet* published the largest number of papers (n=12), followed by *Gynecologic Oncology* (n=11). Of the journals with more than one paper, *The Lancet* had the largest mean number of citations per paper (653.67 citations), followed by *Obstetrics and Gynecology* (577.00 citations) and *The Journal of the National Cancer Institute* (567.80 citations).

**Table 1:** Journals with more than one paper and their impact factors.

Journal Name	Number of Publications	IF (2020)	IF (last five years)
Lancet	12	79.323	77.237
Gynecologic Oncology	11	5.482	5.681
Journal of Clinical Oncology	9	44.544	33.883
Cancer Research	7	12.701	12.843
American Journal of Obstetrics and Gynecology	6	8.661	8.145
New England Journal of Medicine	6	91.253	89.676
Cancer	5	6.860	7.921
Journal of the National Cancer Institute	5	13.506	13.893
Obstetrics and Gynecology	4	7.661	7.248
British Journal of Cancer	3	7.640	7.570
Human Pathology	3	3.466	3.456
Lancet Oncology	3	41.316	44.110
American Journal of Epidemiology	2	4.897	5.827
American Journal of Surgical Pathology	2	6.394	7.507
Annals of Oncology	2	32.976	22.846
Clinical Cancer Research	2	12.531	12.836
Radiology	2	11.105	10.389

#### Countries and institutions

**Figure 3:**
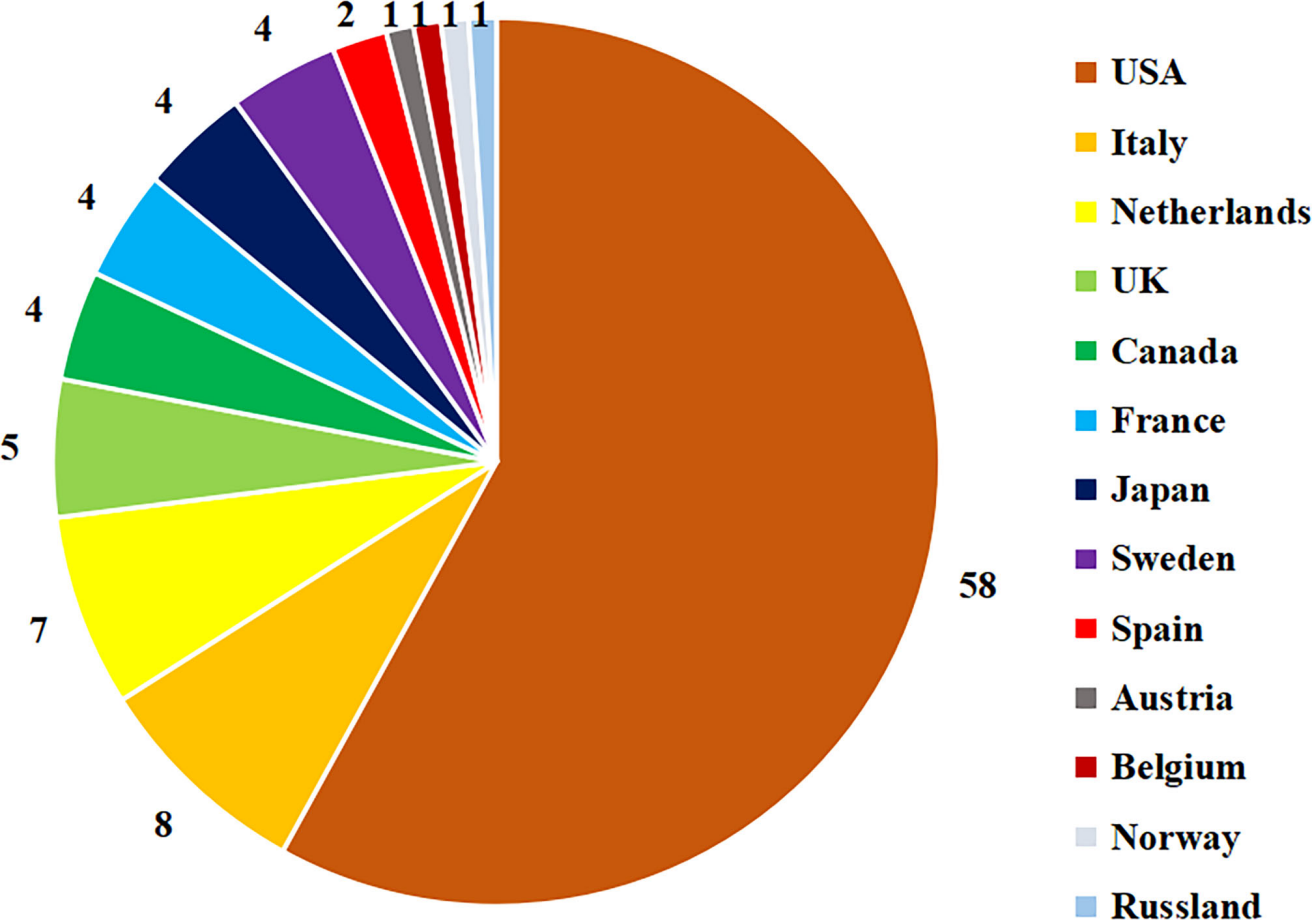
The distribution of countries associated with the top 100 most cited papers.

The corresponding authors of the top 100 most cited papers were from 13 different countries or regions (**Figure 3**). Most of the papers were from the United States (n=58), followed by Italy (n=8) and The Netherlands (n=7). Taking continents into account, North America (n=62) published the most papers, followed by Europe (n=34) and Asia (n=4). The top 100 papers were published from 66 affiliated institutions according to the corresponding author. Duke University, Johns Hopkins University, University of California San Francisco and The University of Southern California (all in United States) contributed the most papers (n=4, respectively). We ranked the institutions with more than one paper in descending order by the number of papers (**Table 2**).

**Table 2:** Institutions with more than one paper in the top 100 most cited papers.

Institution	Record Count
Duke University, Durham, USA	4
Johns Hopkins University, Baltimore, USA	4
University of California, San Francisco, USA	4
University of Southern California, Los Angeles, USA	4
European Institute of Oncology, Milan, Italy	3
Leiden University, Leiden, Netherlands	3
University of Mississippi, Jackson, USA	3
Autonomous University of Barcelona, Barcelona, Spain	2
Harvard University, Boston, USA	2
International Agency for Research on Cancer, Lyon, France	2
Karolinska Institute, Stockholm, Sweden	2
Mayo Clinic, Rochester, USA	2
Medical Research Council Clinical Trials Unit, London, UK	2
Memorial Sloan Kettering Cancer Center, New York, USA	2
Netherlands Cancer Institute, Amsterdam, Netherlands	2
Radcliffe Infirmary, Oxford, UK	2
Stanford University, Stanford, USA	2
University of British Columbia, Vancouver, Canada	2
University of Chicago, Chicago, USA	2
University of North Carolina, Chapel Hill, USA	2
University of Texas, Houston, USA	2
University of Washington, Seattle, USA	2
Yale University, New Haven, USA	2

### Authors

**Figure 4:**
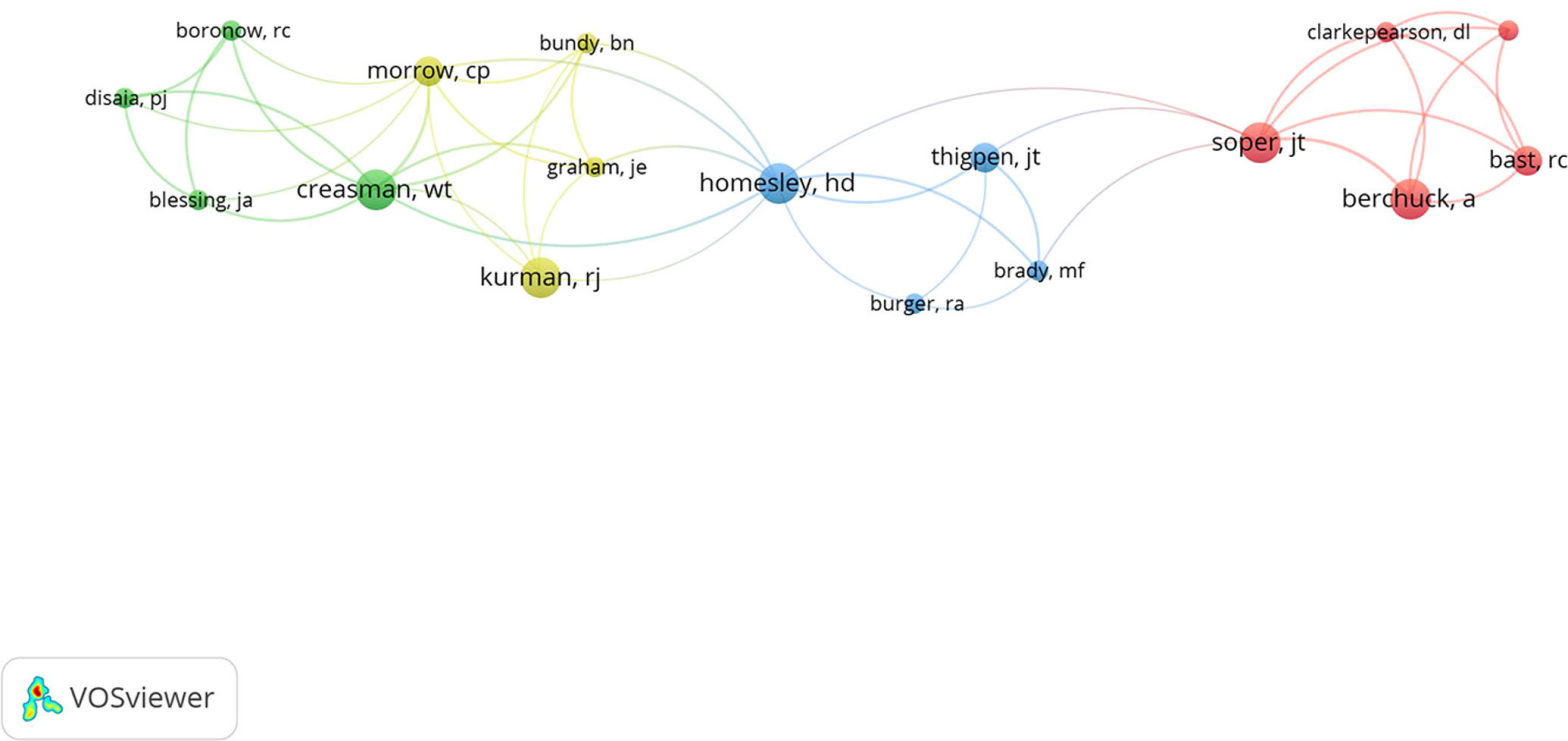
Network visualization map showing the co-authors of the top 100 most cited papers.

A total of 92 authors had been listed as the corresponding author. The corresponding authors with more than one paper are listed in **Table 3**; Nicoletta Colombo from The European Institute of Oncology and University of Milan-Bicocca contributed the most papers (n=3). Then, we constructed a network visualization map for the co-authors of the 100 most cited papers (**Figure 4**). The core of this network was Howard D Homesley, who had the co-authors relationship with at least one author from three other research groups.

**Table 3:** Corresponding authors with more than one paper.

Author Name	Number. of Publications
Colombo, N	3
Creutzberg, CL	2
McAlpine, JN	2
Prat, J	2
Swart, AM	2
Thigpen, JT	2
van Leeuwen, FE	2

### Analysis of research keywords

**Figure 5:**
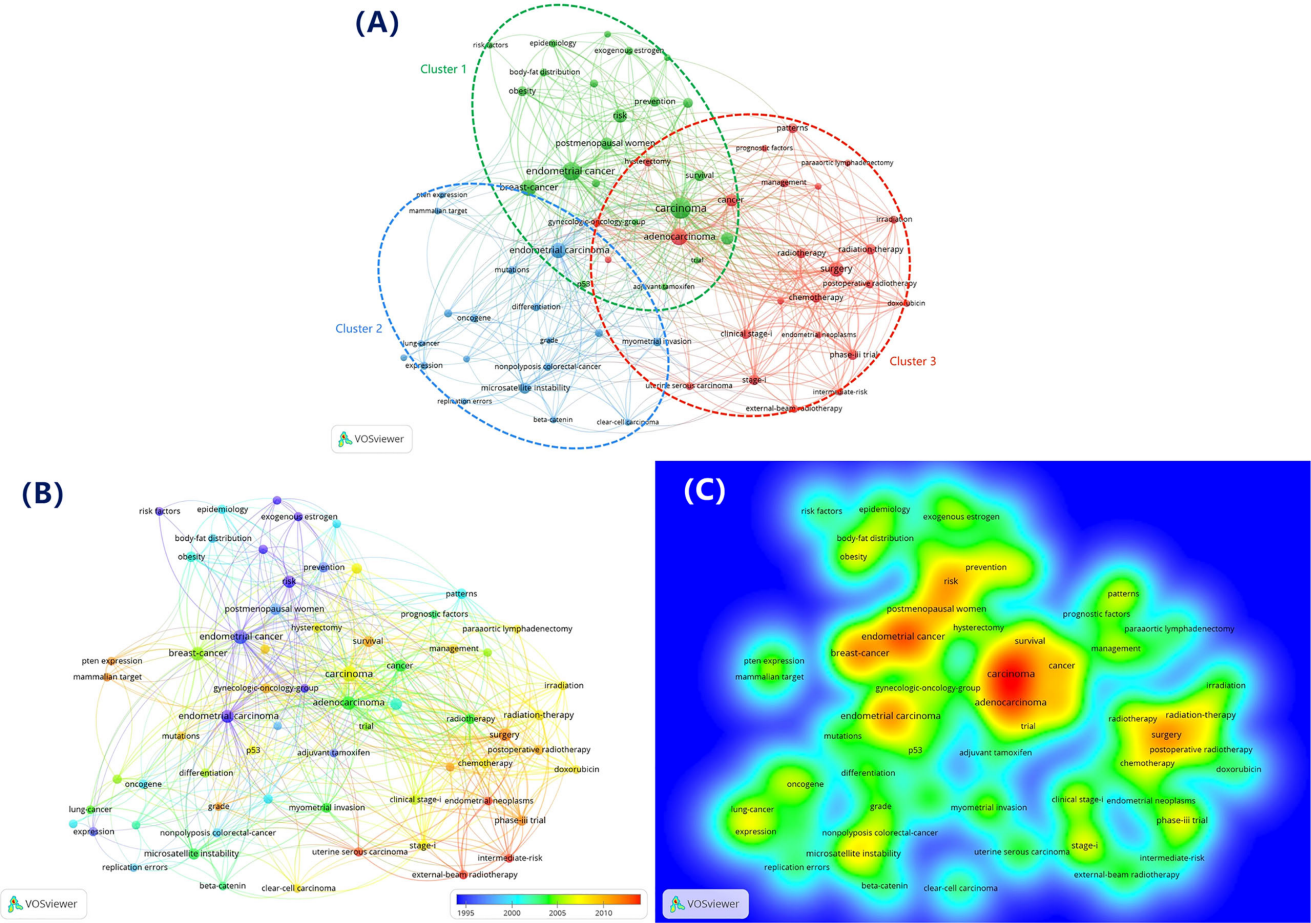
**(A)** Network visualization map showing co-occurrence keywords. **(B)** Overlay visualization map showing co-occurrence keywords based on the mean publication year. **(C)** Density visualization map showing co-occurrence keywords based on the mean frequency of appearance.

VOSviewer identified a total of 487 keywords for the top 100 most cited papers. When limiting the minimum number of occurrences of the keywords to 3, sixty-six items were obtained. As shown in **Figure 5A**, these keywords were classified into three clusters: cluster 1 (epidemiology studies, green circle, 22 items), cluster 2 (molecular biology studies, blue circle, 19 items) and cluster 3 (clinical treatment studies, red circle, 25 items). With regards to cluster 1, the most prominent keywords were breast-cancer, postmenopausal women, risk, obesity and prevention. In cluster 2, the frequently used keywords were microsatellite instability, mutations, oncogene, expression and beta-catenin. Cluster 3 was the largest cluster; the primary keywords in this cluster were surgery, radiotherapy, chemotherapy, external-beam radiotherapy and clinical stage-I.

Finally, we constructed an overlay visualization map for the 66 co-occurrence keywords according to the mean publication year (**Figure 5B**); the purple nodes represent the keywords that appeared comparatively earlier in the time course, while the red nodes reflect those with the most recent occurrence. Early research that was published prior to 2005 in this field was mainly focused on epidemiology and molecular biology; the mean publication year for keywords in cluster 3 (clinical treatment studies) was later than other clusters, which indicated this topic has gained increasing attention recently and is likely to remain a major area of research in the future. Furthermore, the keywords “external-beam radiotherapy,” “uterine serous carcinoma,” and “intermediate-risk” showed relatively later mean publication year and lower mean frequency of occurrence (**Figure 5C**), which may become the new research directions in this field.

## Discussion

Endometrial carcinoma is one of the most common gynecological cancers and researchers have published a multitude of papers in this field. Bibliometric analysis can rank papers according to the number of citations and explore the characteristics of published papers based on detailed and reliable parameters (14–18). Analysis of the most cited papers can provide key information for medical researchers in the field to understand the specific research hotspots and future directions (19, 20). In this bibliometric analysis, we identified the top 100 most cited papers on endometrial carcinoma and analyzed their bibliometric characteristics.

Among the top 100 most cited papers, the sum of citation counts for the top 10 papers accounted for approximately one-third of the total number of citations. “Integrated genomic characterization of endometrial carcinoma” by Getz et al., published in *Nature* in 2013 (21) had the highest number of citations and the highest mean number of citations per year. The number of citations for this paper was significantly higher than any of the other top 10 papers. This particular study used array- and sequencing-based technologies to characterize the integrated genome, transcriptome and proteome of 373 endometrial carcinomas. The results indicated that 25% of high-grade endometrioid tumors and serous tumors had extensive changes in copy number, few DNA methylation alterations, frequent *p53* mutations and low levels of the estrogen and progesterone receptors. The majority of endometrioid tumors had few *TP53* mutations or copy number changes along with novel mutations in the *SWI/SNF* chromatin remodeling complex gene *ARID5B* and frequent mutations in the *CTNNB1*, *KRAS*, *PTEN*, *ARID1A* and *PIK3CA* genes. A subset of the endometrioid tumors identified by the investigators possessed newly identified hotspot mutations with prominently increased transversion mutation frequency in *POLE*. Based on these results, the researchers classified endometrial carcinomas into four categories: *POLE* ultra mutated, microsatellite instability hypermutated, low copy-number and high copy-number. The previous dualist classification had overlapping molecular features, and some cases were not completely consistent with pathological features. This molecular classification method had a direct impact on patient treatment recommendations and provided significant opportunities for genome-based clinical trials and drug discovery (22–24).

Our analysis revealed that the majority of the top 100 most cited papers were published between 1996 and 2000 and between 2006 and 2010. Most of the papers published between 1996 and 2000 were related to the epidemiology and molecular biology of endometrial carcinoma. Of the papers that were specifically related to epidemiology, the risk of endometrial carcinoma was found to be increased following the long-term use of estrogens with or without the cyclically addition of progestins (25–27), especially when administered for 5 years or more. Furthermore, endometrial carcinoma was associated with a poor prognosis following the long-term use of tamoxifen. In addition, women with a positive history of estrogen replacement therapy or those who are obese have an increased risk of endometrial carcinoma when compared to women without a history of tamoxifen (28, 29). However, a diet that is rich in legumes and low in calories, and features whole grain foods, fruits and vegetables may reduce the risk of endometrial carcinoma (30). Of the papers related to molecular biology, mutations in the tumor suppressor gene *PTEN* were more common in endometrial cancers than other known genes; furthermore, this gene plays a vital role in the pathogenesis of endometrioid carcinoma (31, 32). In addition, the hypermethylation of *MLH1* was associated with the *MSI* phenotype in sporadic endometrial carcinoma; *MSH2* was not associated with the *MSI* phenotype but was suggested to play a greater role in genetic susceptibility to endometrial carcinoma (33, 34). The accumulation of beta-catenin and the subsequent activation of the beta-catenin/Tcf pathway may play a significant role in endometrial carcinogenesis due to mutations in exon 3 of beta-catenin, at least in part (35).

In contrast, most of the papers published between 2006 and 2010 were related to clinical treatment. With regards to treatment, pelvic lymphadenectomy did not significantly improve relapse-free or overall survival in patients with endometrial carcinoma (36, 37); instead, combined para-aortic and pelvic lymphadenectomy was recommended for the treatment of patients at medium or high risk of recurrence (38). A high rate of lymphatic metastasis above the inferior mesenteric artery was found to indicate the need for systematic para-aortic and pelvic lymphadenectomy, whereas lymphadenectomy did not provide benefit for patients with grade 1 and 2 endometrioid lesions with a primary tumor diameter less than or equal to 2cm and myometrial invasion less than or equal to 50% (39). Given this information, it was evident that there had been a transformation in the research focus of papers published in the two five-year intervals.

A total of 487 keywords were extracted from the top 100 most cited papers. However, only 13.55% of keywords appeared at least three times, revealing that only a limited number of keywords were frequently used in this field. Bibliometric analysis and co-occurrence visualization maps showed that frequent keywords were generally used to identify research hotspots and future directions in the field (40). By analyzing the network visualization and overlay visualization maps of the co-occurrence keywords of the top 100 papers on endometrial carcinoma, it was evident that most of the keywords related to clinical treatment studies had appeared over recent years. However, most of the keywords in previous papers, especially those published prior to 2005, were related to epidemiological and molecular biology studies. The evident change in keywords over time supported the fact that the clinical treatment of endometrial carcinoma had received increasing levels of attention over recent years. And clinical treatment studies are likely to remain a major area of research in the future. In the future research, scholars still need to further research on genome-based clinical trials and drug discovery. In order to provide more targeted treatment recommendations for patients with different genotypes of endometrial carcinoma.

In terms of research types, the studies related to the clinical treatment in the top 100 most cited papers were primarily conducted through randomized controlled trials which had the highest number of citations on average. Papers using randomized controlled trials were more likely to be published in journals with a higher impact factor than other types of research (36, 37, 41, 42). With regards to other types of research paper, those related to basic science and observational studies had a similar number of citations; the total number of papers related to these two types of research accounted for two-thirds of the 100 most cited papers.

Our bibliometric analysis identified the top six journals with more than one paper, including the top comprehensive journals with the high impact factors such as *The Lancet* and *The New England Journal of Medicine*. These prestigious journals generally publish papers relating to multiple medical disciplines and therefore appeal to a wide range of audiences. *The Lancet* had a total number of 7844 citations and an impact factor of 79.323 in 2020; this journal published the highest number of papers in the top 100 most cited papers and published three papers that had been cited more than 1000 times (36, 43, 44). Due to the subdivision of topics covered, there were more papers on endometrial carcinoma published in specific journals relating to gynecology and oncology, such as *Gynecologic Oncology*, *Journal of Clinical Oncology* and *American Journal of Obstetrics and Gynecology*. In addition, we found that 55% of papers had an impact factor exceeding 10, this may be due to inherent bias in that researchers tend to select journals with high impact factors for citation.

Of the top 100 most cited papers, we identified 92 different corresponding authors from 13 countries. Analysis showed that most authors originated from the United States; this was consistent with bibliometric analyses in other fields, such as head and neck cancer, esophageal cancer and bladder cancer (45–47). In terms of affiliated institutions, Duke University, Johns Hopkins University, University of California San Francisco and University of Southern California were the institutions that contributed the most papers and were all located in the United States. The predominance of academic activity in the United States compared with other countries may be explained by the higher levels of government funding for academic research, a long history of publishing and highly regarded authors. In addition, some researchers have found that reviewers from the United States tend to be more lenient than those from other countries (48). Taking continents into account, North America published the most papers, followed by Europe and Asia. None of the top 100 most cited papers were published in South America, Oceania, Antarctica or Africa. This result is consistent with the highest incidence of endometrial carcinoma in North America and Europe (49).

This is the first study to identify and assess the characteristics of documents related to endometrial carcinoma. Compared with the traditional literature review, the bibliometric analysis by VOSviewer is more comprehensive and objective. However, there are some limitations that need to be considered when interpreting our findings. First, although the Web of Science is a multidisciplinary and comprehensive database covering the field of natural science from across the globe (50), there are still some papers missing. Second, we restricted and filtered our search results; this may have led to some papers being excluded by error. Third, citation analysis does not exclude the influence of self-citation and an author’s preference for specific journals (51, 52) although studies have shown that self-citation only has a minor influence on bibliometric studies (53). Fourth, our ranking of the top 100 most cited papers cannot accurately measure the quality and current research value of papers, because papers published in recent years are less likely to be cited (54, 55). Furthermore, citation rankings may not be used to measure the quality of papers; rather, they only reflect a degree of recognition (56). In the future, we may use multimethod evaluations to gain a more in depth understanding of this research field.

To conclude, this study provides medical researchers with bibliometric information relating to endometrial carcinoma. Our results show that the United States is a clear leader in this field. The clinical treatment of endometrial carcinoma has received increasing levels of attention over recent years and is likely to remain a major area of research in the future. Meanwhile, it is recommended to pay attention to potential research hotspots, such as external-beam radiotherapy, uterine serous carcinoma and intermediate-risk.

## Data availability statement

The original contributions presented in the study are included in the article/**Supplementary Material**. Further inquiries can be directed to the corresponding author.

## Author contributions

GW conceived the study and revised the content of this manuscript. CY made significant contributions to the study methods, results, and interpretation. PX was involved in the design and writing of the manuscript. All authors contributed to the article and approved the submitted version.

## Funding

This work was supported by the Natural Science Foundation of Shandong Province (Grant No. ZR2019MH043).

## Conflict of interest

The authors declare that the research was conducted in the absence of any commercial or financial relationships that could be construed as a potential conflict of interest.

## Publisher’s note

All claims expressed in this article are solely those of the authors and do not necessarily represent those of their affiliated organizations, or those of the publisher, the editors and the reviewers. Any product that may be evaluated in this article, or claim that may be made by its manufacturer, is not guaranteed or endorsed by the publisher.
