# Supplementary material for: The top 100 most cited papers on endometrial carcinoma: A bibliometric analysis
Source: Front Oncol. 2022 Aug 18;12:987980. doi: 10.3389/fonc.2022.987980 (PMC9433873; doi:10.3389/fonc.2022.987980)
Supplement: Supplementary file 1 [file Table_1.docx]

Supplementary Material

**Table S1** The top 100 most cited papers on endometrial carcinoma from 1971 to 2020

| **Rank** | **Title** | **Citation Count** | **Journal** | **Publication Year** | **Corresponding Author** |
| --- | --- | --- | --- | --- | --- |
| 1 | Integrated genomic characterization of endometrial carcinoma | 2487 | Nature | 2013 | Getz G |
| 2 | Surgical pathologic spread patterns of endometrial cancer. A Gynecologic Oncology Group Study | 1628 | Cancer | 1987 | Creasman WT |
| 3 | Two pathogenetic types of endometrial carcinoma | 1452 | Gynecologic Oncology | 1983 | Bokhman JV |
| 4 | Surgery and postoperative radiotherapy versus surgery alone for patients with stage-1 endometrial carcinoma: multicentre randomised trial | 1262 | Lancet | 2000 | Creutzberg CL |
| 5 | Efficacy of systematic pelvic endometrial cancer (MRC ASTEC trial): a randomised study | 1217 | Lancet | 2009 | Swart AM |
| 6 | Relationship between surgical-pathological risk factors and outcome in clinical stage I and II carcinoma of the endometrium: a Gynecologic Oncology Group study | 1099 | Gynecologic Oncology | 1991 | Morrow CP |
| 7 | Endometrial cancer | 1073 | Lancet | 2005 | Neven P |
| 8 | Endometrial cancer in tamoxifen-treated breast cancer patients: findings from the National Surgical Adjuvant Breast and Bowel Project (NSABP) B-14 | 1038 | Journal of The National Cancer Institute | 1994 | Fisher B |
| 9 | Systematic pelvic lymphadenectomy vs. no lymphadenectomy in early-stage endometrial carcinoma: randomized clinical trial | 1033 | Journal of The National Cancer Institute | 2008 | Panici PB |
| 10 | Association of exogenous estrogen and endometrial carcinoma | 933 | New England Journal of Medicine | 1975 | Smith DC |
| 11 | Increased risk of endometrial carcinoma among users of conjugated estrogens | 849 | New England Journal of Medicine | 1975 | Ziel HK |
| 12 | Endometrial cancer | 765 | Lancet | 2016 | Morice P |
| 13 | Obesity, endogenous hormones, and endometrial cancer risk: a synthetic review | 753 | Cancer Epidemiology Biomarkers & Prevention | 2002 | Kaaks R |
| 14 | Hormone replacement therapy and endometrial cancer risk: A meta-analysis | 743 | Obstetrics and Gynecology | 1997 | Grady D |
| 15 | Vaginal brachytherapy versus pelvic external beam radiotherapy for patients with endometrial cancer of high-intermediate risk (PORTEC-2): an open-label, non-inferiority, randomised trial | 701 | Lancet | 2010 | Nout RA |
| 16 | Postoperative external irradiation and prognostic parameters in stage I endometrial carcinoma: clinical and histopathologic study of 540 patients | 698 | Obstetrics and Gynecology | 1980 | Aalders J |
| 17 | Randomized phase III trial of whole-abdominal irradiation versus doxorubicin and cisplatin chemotherapy in advanced endometrial carcinoma: A gynecologic oncology group study | 642 | Journal of Clinical Oncology | 2006 | Randall ME |
| 18 | Mutations in PTEN are frequent in endometrial carcinoma but rare in other common gynecological malignancies | 613 | Cancer Research | 1997 | Tashiro H |
| 19 | Uterine papillary serous carcinoma: a highly malignant form of endometrial adenocarcinoma | 596 | American Journal of Surgical Pathology | 1982 | Hendrickson M |
| 20 | Prospective assessment of lymphatic dissemination in endometrial cancer: a paradigm shift in surgical staging | 569 | Gynecologic Oncology | 2008 | Podratz KC |
| 21 | Estrogens and endometrial cancer in a retirement community | 543 | New England Journal of Medicine | 1976 | Mack TM |
| 22 | Surgical staging in endometrial cancer: clinical-pathologic findings of a prospective study | 541 | Obstetrics and Gynecology | 1984 | Boronow RC |
| 23 | ESMO-ESGO-ESTRO consensus conference on endometrial cancer: diagnosis, treatment and follow-up | 536 | Annals of Oncology | 2016 | Colombo N |
| 24 | Survival effect of para-aortic lymphadenectomy in endometrial cancer (SEPAL study): a retrospective cohort analysis | 504 | Lancet | 2010 | Sakuragi N |
| 25 | Risk of endometrial cancer after tamoxifen treatment of breast cancer | 488 | Lancet | 1994 | van Leeuwen FE |
| 26 | PTEN/MMAC1 mutations in endometrial cancers | 466 | Cancer Research | 1997 | Risinger JI |
| 27 | Endovaginal ultrasound to exclude endometrial cancer and other endometrial abnormalities | 454 | Journal of The American Medical Association | 1998 | Smith-Bindman R |
| 28 | Endometrial cancer and hormone-replacement therapy in the Million Women Study | 430 | Lancet | 2005 | Beral V |
| 29 | Adjuvant external beam radiotherapy in the treatment of endometrial cancer (MRC ASTEC and NCIC CTG EN.5 randomised trials): pooled trial results,, systematic review, and meta-analysis | 424 | Lancet | 2009 | Swart AM |
| 30 | Medical progress: endometrial carcinoma | 422 | New England Journal of Medicine | 1996 | Rose PG |
| 31 | Type I and II endometrial cancers: have they different risk factors? | 411 | Journal of Clinical Oncology | 2013 | Setiawan VW |
| 32 | Effect of obesity on conversion of plasma androstenedione to estrone in postmenopausal women with and without endometrial cancer | 408 | American Journal of Obstetrics and Gynecology | 1978 | MacDonald PC |
| 33 | The dose-effect relationship between 'unopposed' oestrogens and endometrial mitotic rate: its central role in explaining and predicting endometrial cancer risk | 404 | British Journal of Cancer | 1988 | Key TJ |
| 34 | Phase III trial of doxorubicin plus cisplatin with or without paclitaxel plus filgrastim in advanced endometrial carcinoma: A gynecologic oncology group study | 397 | Journal of Clinical Oncology | 2004 | Fleming GF |
| 35 | Risk and prognosis of endometrial cancer after tamoxifen for breast cancer | 387 | Lancet | 2000 | van Leeuwen FE |
| 36 | MLH1 promoter hypermethylation is associated with the microsatellite instability phenotype in sporadic endometrial carcinomas | 385 | Oncogene | 1998 | Herman JG |
| 37 | A comparison of sentinel lymph node biopsy to lymphadenectomy for endometrial cancer staging (FIRES trial): a multicentre, prospective, cohort study | 369 | Lancet Oncology | 2017 | Rossi EC |
| 38 | Endometrial cancer: ESMO Clinical Practice Guidelines for diagnosis, treatment and follow-up | 360 | Annals of Oncology | 2013 | Colombo N |
| 39 | Risk of endometrial cancer in relation to use of oestrogen combined with cyclic progestagen therapy in postmenopausal women | 360 | Lancet | 1997 | Beresford SAA |
| 40 | Endometrial cancer and estrogen use. Report of a large case-control study | 350 | New England Journal of Medicine | 1979 | R Garcia |
| 41 | Detection rate and diagnostic accuracy of sentinel-node biopsy in early stage endometrial cancer: a prospective multicentre study (SENTI-ENDO) | 346 | Lancet Oncology | 2011 | Darai E |
| 42 | High frequency of coexistent mutations of PIK3CA and PTEN genes in endometrial carcinoma | 346 | Cancer Research | 2005 | McCormick F |
| 43 | The accuracy of endometrial sampling in the diagnosis of patients with endometrial carcinoma and hyperplasia – a meta-analysis | 341 | Cancer | 2000 | Dijkhuizen FPHLJ |
| 44 | Risk of endometrial cancer after treatment with oestrogens alone or in conjunction with progestogens: results of a prospective study | 340 | British Medical Journal | 1989 | Persson I |
| 45 | Diabetes mellitus and risk of endometrial cancer: a meta-analysis | 337 | Diabetologia | 2007 | Friberg E |
| 46 | Beta-catenin mutation in carcinoma of the uterine endometrium | 335 | Cancer Research | 1998 | Hirohashi S |
| 47 | Association of polymerase e-mutated and microsatellite-instable endometrial cancers with neoantigen load, number of tumor-infiltrating lymphocytes, and expression of PD-1 and PD-L1 | 328 | JAMA Oncology | 2015 | Konstantinopoulos PA |
| 48 | Classification of endometrial carcinoma: more than two types | 327 | Lancet Oncology | 2014 | Murali R |
| 49 | Randomized phase III trial of pelvic radiotherapy versus cisplatin-based combined chemotherapy in patients with intermediate- and high-risk endometrial cancer: A Japanese Gynecologic Oncology Group study | 327 | Gynecologic Oncology | 2008 | Sagae S |
| 50 | High-grade endometrial carcinoma in tamoxifen-treated breast cancer patients | 327 | Journal of Clinical Oncology | 1993 | Magriples U |
| 51 | Retrospective analysis of selective lymphadenectomy in apparent early-stage endometrial cancer | 326 | Journal of Clinical Oncology | 2005 | Havrilesky LJ |
| 52 | Progestin treatment of atypical hyperplasia and well-differentiated carcinoma of the endometrium in women under age 40 | 326 | Obstetrics and Gynecology | 1997 | Randall TC |
| 53 | The epidemiology of endometrial cancer | 326 | Gynecologic Oncology | 1991 | Parazzini F |
| 54 | Concurrent endometrial carcinoma in women with a biopsy diagnosis of atypical endometrial hyperplasia - A Gynecologic Oncology Group Study | 325 | Cancer | 2006 | Trimble CL |
| 55 | Oral medroxyprogesterone acetate in the treatment of advanced or recurrent endometrial carcinoma: A dose-response study by the Gynecologic Oncology Group | 317 | Journal of Clinical Oncology | 1999 | Thigpen JT |
| 56 | Sequential adjuvant chemotherapy and radiotherapy in endometrial cancer - Results from two randomised studies | 314 | European Journal of Cancer | 2010 | Hogberg T |
| 57 | Adjuvant chemotherapy vs radiotherapy in high-risk endometrial carcinoma: results of a randomised trial | 314 | British Journal of Cancer | 2006 | Fossati R |
| 58 | m(6)A mRNA methylation regulates AKT activity to promote the proliferation and tumorigenicity of endometrial cancer | 310 | Nature Cell Biology | 2018 | He C |
| 59 | The G protein-coupled receptor GPR30 mediates the proliferative effects induced by 17 beta-estradiol and hydroxytamoxifen in endometrial cancer cells | 302 | Molecular Endocrinology | 2006 | Maggiolini M |
| 60 | Search for cancer markers from endometrial tissues using differentially labeled tags iTRAQ and clCAT with multidimensional liquid chromatography and tandem mass spectrometry | 302 | Journal of Proteome Research | 2005 | Siu KWM |
| 61 | High frequency of PIK3R1 and PIK3R2 mutations in endometrial cancer elucidates a novel mechanism for regulation of PTEN protein stability | 300 | Cancer Discovery | 2011 | Cheung LWT |
| 62 | Risk of endometrial cancer following estrogen replacement with and without progestins | 299 | Journal of the National Cancer Institute | 1999 | Weiderpass E |
| 63 | A clinically applicable molecular-based classification for endometrial cancers | 298 | British Journal of Cancer | 2015 | McAlpine JN |
| 64 | Risk factors and recurrent patterns in Stage I endometrial cancer | 297 | American Journal of Obstetrics and Gynecology | 1985 | DiSaia PJ |
| 65 | ESMO-ESGO-ESTRO consensus conference on endometrial cancer diagnosis, treatment and follow-up | 294 | International Journal of Gynecological Cancer | 2016 | Colombo N |
| 66 | Primary uterine endometrial stromal neoplasms. A clinicopathologic study of 117 cases | 293 | American Journal of Surgical Pathology | 1990 | Chang KL |
| 67 | Radiologic staging in patients with endometrial cancer: a meta-analysis | 287 | Radiology | 1999 | Hricak H |
| 68 | Elevation of serum CA125 in carcinomas of the fallopian tube, endometrium, and endocervix | 279 | American Journal of Obstetrics and Gynecology | 1984 | Niloff JM |
| 69 | Drug-sensitive FGFR2 mutations in endometrial carcinoma | 278 | Proceedings of The National Academy of Sciences of The United States of America | 2008 | Meyerson M |
| 70 | Definition of microRNAs that repress expression of the tumor suppressor gene FOXO1 in endometrial cancer | 277 | Cancer Research | 2010 | Lam EWF |
| 71 | Overexpression and mutation of p53 in endometrial carcinoma | 277 | Cancer Research | 1992 | Kohler MF |
| 72 | Molecular genetic pathways in various types of endometrial carcinoma: from a phenotypical to a molecular-based classification | 276 | Virchows Archiv | 2004 | Lax SF |
| 73 | Replacement estrogens and endometrial cancer | 275 | New England Journal of Medicine | 1979 | Jick H |
| 74 | p53 in endometrial cancer and its putative precursors: evidence for diverse pathways of tumorigenesis | 271 | Human Pathology | 1995 | Sherman ME |
| 75 | Improved risk assessment by integrating molecular and clinicopathological factors in early-stage endometrial cancer-combined analysis of the PORTEC cohorts | 270 | Clinical Cancer Research | 2016 | Bosse T |
| 76 | Comparison of outcomes and cost for endometrial cancer staging via traditional laparotomy, standard laparoscopy and robotic techniques | 268 | Gynecologic Oncology | 2008 | Bell MC |
| 77 | Multicenter phase II study of fertility-sparing treatment with medroxyprogesterone acetate for endometrial carcinoma and atypical hyperplasia in young women | 260 | Journal of Clinical Oncology | 2007 | Ushijima K |
| 78 | Exogenous estrogen and endometrial carcinoma: case-control and incidence study | 260 | American Journal of Obstetrics and Gynecology | 1977 | McDonald TW |
| 79 | Phase III trial of doxorubicin with or without cisplatin in advanced endometrial carcinoma: A gynecologic oncology group study | 259 | Journal of Clinical Oncology | 2004 | Thigpen JT |
| 80 | Survival after relapse in patients with endometrial cancer: results from a randomized trial | 259 | Gynecologic Oncology | 2003 | Creutzberg CL |
| 81 | Intraabdominal lymphatic mapping to direct selective pelvic and paraaortic lymphadenectomy in women with high-risk endometrial cancer: results of a pilot study | 255 | Gynecologic Oncology | 1996 | Burke TW |
| 82 | Reproductive, menstrual, and medical risk factors for endometrial cancer: results from a case-control study | 255 | American Journal of Obstetrics and Gynecology | 1992 | Brinton LA |
| 83 | The PI3K/AKT/mTOR Pathway as a therapeutic target in endometrial cancer | 251 | Clinical Cancer Research | 2012 | Slomovitz BM |
| 84 | Evaluation of criteria for distinguishing atypical endometrial hyperplasia from well-differentiated carcinoma | 250 | Cancer | 1982 | Kurman RJ |
| 85 | Confirmation of ProMisE: a Simple, genomics-based clinical classifier for endometrial cancer | 248 | Cancer | 2017 | McAlpine JN |
| 86 | Metformin is a potent inhibitor of endometrial cancer cell proliferation-implications for a novel treatment strategy | 246 | Gynecologic Oncology | 2010 | Bae-Jump VL |
| 87 | Molecular pathology of endometrial hyperplasia and carcinoma | 244 | Human Pathology | 2001 | Prat J |
| 88 | Association of soy and fiber consumption with the risk of endometrial cancer | 242 | American Journal of Epidemiology | 1997 | Goodman MT |
| 89 | The importance of applying a sentinel lymph node mapping algorithm in endometrial cancer staging: Beyond removal of blue nodes | 241 | Gynecologic Oncology | 2012 | Abu-Rustum NR |
| 90 | Overexpression of HER-2/neu in endometrial cancer is associated with advanced stage disease | 240 | American Journal of Obstetrics and Gynecology | 1991 | Berchuck A |
| 91 | Local-regional staging of endometrial carcinoma: role of MR imaging in surgical planning | 239 | Radiology | 2004 | Manfredi R |
| 92 | Tamoxifen therapy for breast cancer and endometrial cancer risk | 238 | Journal of the National Cancer Institute | 1999 | Bernstein L |
| 93 | A case-control study of cancer of the endometrium | 237 | American Journal of Epidemiology | 1982 | Kelsey JL |
| 94 | Phase II study of temsirolimus in women with recurrent or metastatic endometrial cancer: a trial of the NCIC clinical trials group | 235 | Journal of Clinical Oncology | 2011 | Oza AM |
| 95 | Progestagen supplementation of exogenous oestrogens and risk of endometrial cancer | 233 | Lancet | 1991 | Voigt LF |
| 96 | International patterns and trends in endometrial cancer incidence, 1978-2013 | 231 | Journal of the National Cancer Institute | 2018 | Lortet-Tieulent J |
| 97 | Prognostic parameters of endometrial carcinoma | 231 | Human Pathology | 2004 | Prat J |
| 98 | MLH1 promoter methylation and gene silencing is the primary cause of microsatellite instability in sporadic endometrial cancers | 230 | Human Molecular Genetics | 1999 | Goodfellow PJ |
| 99 | Endometrial cancer and obesity: epidemiology, biomarkers, prevention and survivorship | 228 | Gynecologic Oncology | 2009 | von Gruenigen VE |
| 100 | hCDC4 gene mutations in endometrial cancer | 228 | Cancer Research | 2002 | Reed SI |
